# Monitoring adverse social and medical events in public health trials: assessing predictors and interpretation against a proposed model of adverse event reporting

**DOI:** 10.1186/s13063-019-3961-8

**Published:** 2019-12-30

**Authors:** Gwenllian Moody, Katy Addison, Rebecca Cannings-John, Julia Sanders, Carolyn Wallace, Michael Robling

**Affiliations:** 1 Centre for Trials Research, Cardiff University, Neuadd Meirionnydd, Heath Park, Cardiff, UK; 2 School of Healthcare Sciences, Cardiff University, Heath Park, Cardiff, UK; 3 PRIME (Wales Centre for Primary and Emergency Care Research), Heath Park, Cardiff, UK

**Keywords:** Safety monitoring, Adverse event, Serious adverse event, Public health, Clinical trials, Home visiting

## Abstract

**Background:**

Although adverse event (AE) monitoring in trials focusses on medical events, social outcomes may be important in public or social care trials. We describe our approach to reporting and categorising medical and other AE reports, using a case study trial. We explore predictors of medical and social AEs, and develop a model for conceptualising safety monitoring.

**Methods:**

The Building Blocks randomised controlled trial of specialist home visiting recruited 1618 first-time mothers aged 19 years or under at 18 English sites. Event reports collected during follow-up were independently reviewed and categorised as either Medical (standard Good Clinical Practice definition), or Social (trial-specific definition). A retrospectively developed system was created to classify AEs. Univariate analyses explored the association between baseline participant and study characteristics and the subsequent reporting of events. Factors significantly associated at this stage were progressed to binary logistic regressions to assess independent predictors.

**Results:**

A classification system was derived for reported AEs that distinguished between Medical or Social AEs. One thousand, three hundred and fifteen event reports were obtained for mothers or their babies (1033 Medical, 257 Social). Allocation to the trial intervention arm was associated with increased likelihood of Medical rather than Social AE reporting. Poorer baseline psycho-social status predicted both Medical and Social events, and poorer psycho-social status better predicted Social rather than Medical events. Baseline predictors of Social AEs included being younger at recruitment (OR = 0.78 (CI = 0.67 to 0.90), *p* = 0.001), receiving benefits (OR = 1.60 (CI = 1.09 to 2.35), *p* = 0.016), and having a higher antisocial behaviour score (OR = 1.22 (CI = 1.09 to 1.36), *p* < 0.001). Baseline predictors of Medical AEs included having a limiting long-term illness (OR = 1.37 (CI = 1.01 to 1.88), *p* = 0.046), poorer mental health (OR = 1.03 (CI = 1.01 to 1.05), *p* = 0.004), and being in the intervention arm of the trial (OR = 1.34 (CI = 1.07 to 1.70), *p* = 0.012).

**Conclusions:**

Continuity between baseline and subsequent adverse experiences was expected despite potentially beneficial intervention impact. We hypothesise that excess events reported for intervention-arm participants is likely attributable to surveillance bias. We interpreted our findings against a new model that explicates processes that may drive event occurrence, presentation and reporting. Focussing only upon Medical events may miss the well-being and social circumstances that are important for interpreting intervention safety and participant management.

**Trial registration:**

ISRCTN, ID: ISRCTN23019866. Registered on 20 April 2009.

## Background

Adverse event (AE) reporting is an integral part of safety monitoring for clinical trials. However, the processes for collecting, recording, analysing, and reporting AEs can be considered to be more complex and less developed than those processes used when evaluating efficacy in a trial [1]. Safety monitoring in clinical trials has been standardised using AE and serious adverse event (SAE) reporting protocols; for example, The Medicines for Human Use (Clinical Trials) Regulations 2004, which focus on medical events of varying severity. Such AEs may or may not be associated with the intervention. In comparison to clinical trials of medicinal products, public health or social care trials will often evaluate complex interventions in populations with adverse social circumstances; for example, in deprived populations. Such interventions may still have unexpected and unwelcome effects. Monitoring unintended or unexpected outcomes in such trials and participant well-being in general will involve outcomes which are social and psychological in nature in addition to medical. Systems for monitoring these events are underdeveloped and inconsistent in public health, social care, and psychotherapy trials; for example, Duggan et al. (2014) found that the recording of AEs in a trial of psychological intervention was either not attempted/reported, or used definitions not entirely suitable to the intervention or condition being studied [2]. While some authors have attempted to expand on the Good Clinical Practice (GCP) definition of AEs and SAEs to incorporate other types of events [3–6], none of these have included social events.

The Building Blocks randomised controlled trial evaluated the effectiveness and cost-effectiveness of the Family Nurse Partnership (FNP) home-visiting programme in England [7, 8]. Field and office-based researchers were responsible for the reporting of AEs on a site level to the trial team.

Monitoring events in the trial performed two functions. The first was to detect any undesirable consequence of the intervention. FNP is a supportive and voluntary home-visiting intervention which was not expected to produce harm, but with up to 64 home visits to women in often vulnerable circumstances, the intensive and structured approach could have been unwelcome to some families. The second purpose was to monitor in general the well-being of research participants in both trial arms. This included attempting to ensure that research processes did not add to participants’ distress if they were experiencing adverse social circumstances, and to facilitate optimal trial processes.

The value of monitoring AEs in trials is in detecting harmful effects attributable to an intervention. However, this signal may be obscured by other non-relevant factors that introduce unhelpful ‘noise’. For example, some studies have found reporting rates of AEs to vary by country [3], by reporter (e.g. clinician vs. participant) [9], and by reporting site. Reporting of AEs by health professionals may depend upon their awareness of the event, their judgement about the event, and their willingness to document the event [10]. Variation in reporting AEs driven by under-developed monitoring systems or inconsistent training reduces the potential to adequately monitor unintended effects of both public health and other interventions.

In summary, systems for AE monitoring in interventional studies in public health and social care are under-developed and variation in reports may be due to factors other than the intervention itself. In this paper our first aim is to describe our approach to reporting and categorising Medical and other AE reports in a large public health trial. Our second aim is to assess variability in safety reporting, and explore factors associated with the nature (i.e. the type of event reported), level (i.e. the level of seriousness) and quality of reporting (for example, any differences between study sites) in our study sample.

## Methods

The Building Blocks trial evaluated the effectiveness of the FNP programme. The intervention consisted of up to 64 home visits from a specially trained Family Nurse during pregnancy and in the 2 years after birth, with the aim of improving outcomes for the health, well-being and social circumstances of young, first-time mothers and their children. The intervention covered core content areas of personal and environmental health, life course development, maternal rôle, family and friends and access to health and social services, including promoting healthy behaviours. The control group did not receive the intervention and instead received usual services, this included the Healthy Child Programme (universally offered screening, education, immunisation, and support from birth to the child’s second birthday) delivered by specialist community public health nurses, and maternity care appropriate to clinical need. Following birth the control group continued to receive postnatal midwifery care and care from existing child health services available locally, including an allocated health visitor. Details of the intervention and control conditions, as well as the full Building Blocks trial methods, can be found in the trial protocol and results papers [7, 8]. Trial outcome data was collected during face-to-face interviews by local researchers and through telephone interviews by staff located in Cardiff who were also responsible for the reporting of AEs to the trial team. From the outset, while a primary focus for safety monitoring was on Medical AEs, other concerns could have been noted by both field and office-based researchers. The collection of AEs was also intended to monitor the general well-being of research participants in both trial arms. For example, we intended to collect information to allow the trial team to have prior knowledge if they were contacting participants at difficult times (e.g. if either a mother or child was undergoing formal safeguarding procedures). Similarly, during the 24-month follow-up interview, scoring positively for items indicating serious abuse on a domestic abuse scale also triggered the completion of an AE form [11]. Detection of domestic abuse via this scale triggered the family’s health visitor being informed, and if ongoing and a new disclosure, resulted in a mandatory referral to social services.

Participants: participants in the Building Blocks trial were 1618 women aged 19 years or under at recruitment and expecting their first child. Young maternal age was used as a programme proxy for a range of poor longer-term outcomes for both child and mother and is also associated with socioeconomic deprivation. It was expected that many trial participants would face challenging individual personal and social circumstances. Baseline characteristics of the participants were collected through a home-based interview prior to randomisation.

Setting: 18 sites in England each comprising partnerships between primary healthcare organisations and local authorities for the purposes of delivering the FNP programme.

Adverse event reporting: AEs were reported during the approximately 2.5-year follow-up period by field and office-based researchers. Field researchers were usually trained midwives or nurses. They collected trial information on outcomes from medical notes as well as in face-to-face interviews (at baseline and final 24 months’ follow-up). They also had a remit to maintain contact with participants for the purposes of data collection. The office-based researchers collected self-report data via telephone interview at late pregnancy, 6, 12 and 18 months following birth. In both telephone and face-to-face interviews AE reports were triggered by participant responses to other open-ended questions or were reported directly from a participant un-prompted. AEs could also be reported by any other health professional associated with the trial including Family Nurses (intervention group only) and general practitioners (GPs). To report AEs a form was completed and sent to the trial team via secure fax, or emailed to the Data Manager. The Building Blocks trial Manager or the Chief Investigator and one clinical member of the research team jointly assessed each form to ascertain the nature, seriousness, causality and expectedness of the AE. Following receipt of the initial form, the trial team could request follow-up data from the reporting site or researcher. Some pregnancy-related events, such as hospitalisation due to child birth, and termination of pregnancy for foetal anomaly, were expected in the context of the trial and were, therefore, not expected to be reported as AEs.

Training: prior to the start of recruitment, field and office-based researchers were trained to collect AEs using a standardised reporting form and following GCP guidance. Instructions were included in the data collection forms (e.g. for the telephone interviews), that reminded interviewers to enquire about participant well-being at the start of the interview (as an open question). Any issues related to well-being at this stage would have been reported as AEs if appropriate to do so. After variations in AE reporting rates were observed during the course of the trial follow-up, additional face-to-face training was provided to all field researchers.

### Aim 1: classifying and coding AEs

For the current analyses we retrospectively developed a system to classify reported AEs. The Chief Investigator (MR), Trial Manager (EO-J), Data Manager (GM), Senior Clinical Researcher (JS), a clinical co-investigator on the Building Blocks trial (JK), and a clinically qualified qualitative researcher (CW) met to develop a classification system following some iterative discussions and review of a sample of submitted AE forms.

Developing the classification: the GCP definitions of AEs and SAEs were used to initially classify forms. A distinction was then made between physical and mental GCP AEs and SAEs as the trial team was interested in distinguishing between participants’ mental and physical well-being. Events that did not fit under the GCP definitions but were considered of particular relevance to the trial were then classified as ‘Social AEs’. These included safeguarding issues, information related to the child being fostered or adopted which in these circumstances may be a proxy for adversity [12], incidents of violence or aggression towards Family Nurses or field researchers, and issues that would be important for researchers to know about before speaking to a participant, such as social circumstances (both at baseline and any changes during the course of the trial), and instances when a participant scored positively for serious abuse on the domestic abuse scale. Events that were recorded on AE forms but did not meet the criteria for any of the above categories were classified as ‘Other events’.

Defining unique events: during classification it was important to define what constituted a discrete ‘event’ as some forms were essentially updates to previous reports. An event was defined as starting from the point of presentation, and continued to be consistently the same ‘condition’ until the end of the event. The end of the event was defined as when the participant had been either discharged from hospital, there was no further attendance or visit required, or no follow-up form was sent. When forms were sent in relation to the same event, the first form sent (by date) was classified, and the rest of the forms were marked as ‘follow-up’. All forms related to the same event were reviewed before classifying an event as ‘follow-up’ as any form could include details that would change an event’s classification. If this was the case the rater would then classify the event using the most serious classification, and thus these events were analysed on the basis of the greater degree of severity. Where more than one event was reported on a form, each event was classified separately.

Coding forms: after the final classification system was agreed, the AE forms were coded by a clinically qualified qualitative researcher (CW) from outside the research team but who had been involved in developing the classification system. A second rater (GM) coded a 10% random selection of events to ascertain reliability of the classification system using Cohen’s Kappa [13].

### Aim 2: exploring sources of variation in rate of reporting AEs

We hypothesised that:

- Poorer psycho-social status and health at baseline will be associated with higher reported rates of both Medical and Social AEs (baseline variables thought to reflect poorer psycho-social status listed below)
- Poorer psycho-social status at baseline will more likely be associated with Social rather than Medical AEs
- AEs reports will be more likely for those in the trial intervention arm (hypothesised to be due to surveillance bias, having received up to 64 visits from a family nurse)
  Rate of AE reporting will vary by trial site (due to various system level differences between sites which could include variability in research nurse approach; e.g. actual funded time, total number of participants at site being monitored, quality of links to local Family Nurses or other local staff). Site was a predictor we sought to modify during the course of the trial, but despite our efforts differences in site were not eradicated.

Baseline variables that we considered to indicate poorer psycho-social status were younger age at recruitment, the woman’s status being classified as NEET (Not in Education, Employment, or Training), being in receipt of benefits, having ever been homeless, having lower socio-economic status (Index of Multiple Deprivation score), lower family and lower personal subjective social status, lower relationship quality, lower social support, lower family resources, lower self-efficacy, and lower adaptive functioning.

All participants were categorised as having experienced either no, or at least one Social AE. They were also categorised as having experienced either no or at least one Medical AE (regardless of severity). These formed the two dependent variables in subsequent analyses. For each dependent variable the following sets of analyses were performed. Baseline characteristics were summarised between those who experienced either no or at least one AE (Social and Medical) using number (%), mean alongside standard deviation (SD) and median alongside the 25th to 75th centiles. Baseline characteristics included socio-demographics listed above, e.g. age; health (e.g. health status, psychological distress) and group allocation. Logistic regression models were run to examine univariable associations between baseline characteristics and AEs. Baseline characteristics that were associated at the 10% significance level were retained and entered as candidate predictors for the multivariable model to detect all characteristics independently predictive based on a significance level of 0.05 of AEs. Trial site was adjusted for by its inclusion as a random effect in all models. Multi-collinearity in each model between candidate predictors was assessed by detecting the tolerance and its reciprocal, the Variance Inflation Factor (VIF). As a rule of thumb a VIF of 1 indicates no collinearity but a VIF greater than 4 (a tolerance of 0.2) might warrant further investigation and greater than 10 would indicate that multi-collinearity is a problematic.

## Results

### Aim 1: classification system for reported AEs

**Fig. 1:**
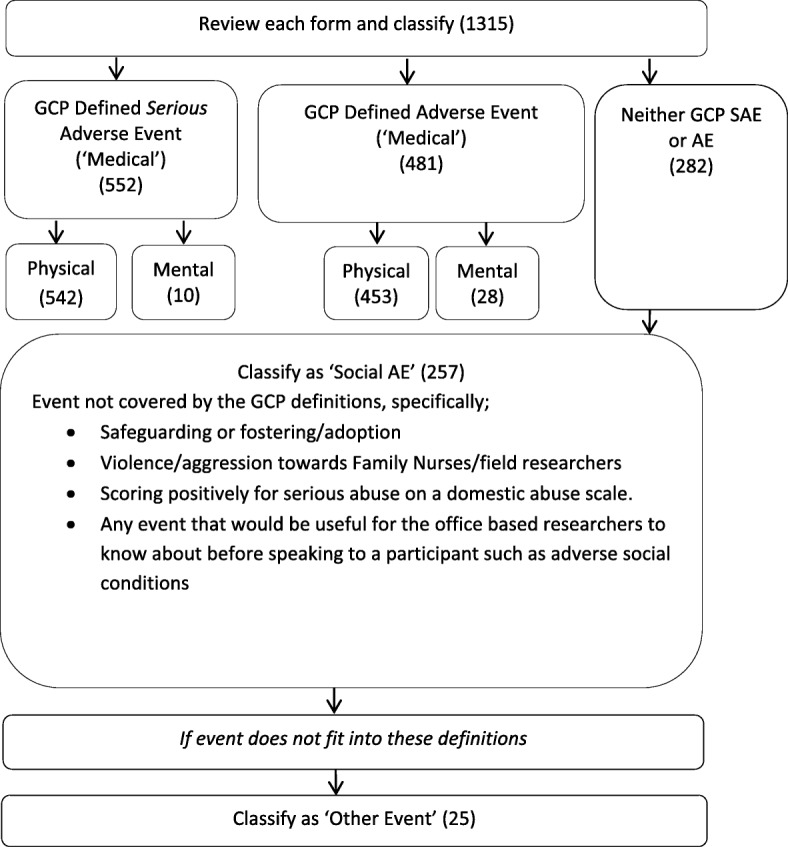
Adverse event (AE) classification in the Building Blocks trial

A classification system was derived for reported AEs (Fig. 1). This distinguished between Medical AEs and Social AEs. The former were further classified into Physical or Mental and by severity (i.e. whether or not serious, severity was determined following the GCP definition). Social AEs encompassed several distinct categories, such as safeguarding, but did not further distinguish between severity. The reliability of coding reports to the classification system was high (Table 1) with an overall Cohen’s Kappa [13] rating of 0.925. Of the 1315 uniquely reported events, 78.6% were Medical AEs (552 SAEs, 481 AEs), 19.5% were coded as Social AEs and a further 25 (1.9%) were coded as ‘Other’ events.

**Table 1:** Reliability of Building Blocks adverse event (AE) classification system

Form classification	Agreement (Cohen’s Kappa [13])
SAE Physical	0.923
SAE Mental	135/137 = 99%^a^
AE Physical	0.950
AE Mental	0.717
Social AE	0.936
Other event	136/137 = 99%^a^
Total	0.925

^a^cell counts were too low to calculate a Kappa. S*AE* serious adverse event

The number of unique events reported by trial site and their classification, whether the event was related to the mother or baby, source of notification, and trial arm are described in the following paragraphs.

One thousand, three hundred and fifteen completed forms were sent to the trial team, relating to 667/1618 (41.2%) participants (or their baby(ies)). The number of events per participant varied considerably from 0 to 27. On average, 0.81 events were reported for each participant (Table 2). For Physical SAEs the rates of reported events ranged from 0.07 to 1.53 per participant (a more than 20-fold difference in similarly sized trial sites).

**Table 2:** Number of events per participant within each site

		Number of events per participant by site and classification of event						
Site ID	Number of participants per site	Physical SAEs	Mental SAEs	Physical AEs	Mental AEs	Social AEs	Other events	Total (all classifications)
1	43	0.91	0.02	1.35	0.05	0.26	0.05	2.63
2	49	0.53	0	0.49	0	0.04	0	1.06
4	143	0.14	0	0.10	0.01	0.08	0.01	0.35
5	39	0.33	0	0.38	0	0.30	0.03	1.04
7	54	0.07	0.02	0.02	0	0.17	0.02	0.30
8	39	0.26	0	0	0.05	0.03	0	0.33
9	47	0.32	0	0.21	0	0.23	0.02	0.79
10	113	0.57	0.02	0.19	0.02	0.09	0	0.88
21	115	0.12	0	0	0	0.12	0.01	0.25
22	68	1.53	0.03	1.77	0.06	0.42	0.04	3.85
23	99	0.19	0.02	0.33	0	0.04	0	0.59
24	93	0.19	0	0.05	0.01	0.18	0.01	0.45
25	150	0.31	0.01	0.07	0	0.17	0.02	0.59
26	35	0.09	0	0	0	0.03	0	0.11
27	142	0.27	0	0.42	0.03	0.08	0	0.80
28	139	0.35	0.01	0.11	0.05	0.41	0.04	0.97
29	117	0.23	0	0.03	0.03	0.15	0.02	0.47
30	133	0.23	0	0.45	0.01	0.09	0.01	0.80
Total	1618	0.33	0.01	0.28	0.02	0.16	0.01	0.81

*AE* adverse event, *SAE* serious adverse event

None of the Social AEs were related to violence or aggression towards Family Nurse or Researchers (as self-reported by the professionals), and most events related to safeguarding (Table 3).

**Table 3:** Details of events classified as Social adverse events (AEs)

Social AE detail (primary reason)	Number of events (%)	Intervention	Control
Adoption/fostering	19 (7.4%)	10 (7.8%)	9 (7.0%)
Composite Abuse Scale identified serious domestic abuse during 2-year interview	33 (12.8%)	13 (10.1%)	20 (15.6%)
Domestic abuse	35 (13.6%)	17 (13.2%)	18 (14.1%)
Safeguarding of mother or child	168 (65.4%)	87 (67.4%)	81 (63.3%)
Social conditions	2 (0.8%)	2 (1.6%)	0 (0.0%)
Total	257	129	128

Events related to mothers accounted for 36.7%, events related to baby(ies) accounted for 42.7%, and events related to both mother and baby(ies) accounted for 20.6%. 614/1315 (46.69%) of events were recorded as being before the birth of the Building Blocks baby(ies).

Over 90% of events were reported by field and office-based researchers as opposed to other health professionals involved with the trial (Table 4).

**Table 4:** Source of event notification

Notifier	Total number of events reported (%)	Intervention	Control
Field researcher	1125 (85.5%)	603 (83.5%)	522 (88.0%)
Office-based researcher	75 (5.7%)	32 (4.4%)	43 (7.3%)
Family nurse	68 (5.1%)	67 (9.3%)	1 (0.2%)
Health visitor	14 (1.1%)	0 (0.0%)	14 (2.4%)
Midwife	5 (0.4%)	2 (0.3%)	3 (0.5%)
GP	1 (0.1%)	0 (0.0%)	1 (0.2%)
Other medical professional	1 (0.1%)	0 (0.0%)	1 (0.2%)
Unknown job rôle	1 (0.1%)	1 (0.1%)	0 (0.0%)
No name on form	25 (1.9%)	17 (2.4%)	8 (1.3%)
Total	1315	722	593

After variations in AE reporting rates were observed during the course of the trial follow-up, additional face-to-face training was provided on two dates to all field researchers. The number of events reported before the first training day was 1030 (78.3%), the number of events reported between the two training dates (including first training date) was 14 (1.1%), and the number of events reported post training (including the second training date) was 109 (8.3%); 162 (12.3%) events were reported that did not contain an event date. The referenced here is event date, rather than reporting date; therefore, caution should be taken as the event may have taken place sometime before it was reported.

### Aim 2: analysis of variation in rate of reporting AEs

Baseline characteristics were compared for participants with and without at least one Social AE (Table 5) and for participants with and without at least one Medical event (either AE or SAE) (Table 6).

**Table 5:** Baseline characteristics of participants with and without at least 1 Social adverse event (AE)

	At least 1 Social AE *N* = 208		Participants without a Social AE *N* = 1410		Unadjusted OR^a^ (95% CI), *p* value	Adjusted OR^b^ (95% CI), *p* value
	*n*	Median (25th to 75th centile) or %	*n*	Median (25th to 75th centile) or %		
Baseline characteristic						
Maternal age (years)	208	17.6 (16.7 to 18.6)	1410	17.9 (17.0 to 18.8)	***0.84 (0.74 to 0.94), 0.004***	***0.78 (0.67 to 0.90), 0.001***
Ethnicity					Overall *p* value = 0.189	
White background	190	91.3	1235	87.6	Ref	
Mixed background	13	6.2	76	5.4	1.07 (0.57 to 2.02), 0.834	
Asian background	3	1.4	24	1.7	0.59 (0.15 to 2.26), 0.439	
Black background	2	1.0	69	4.9	0.22 (0.05 to 0.95), 0.042	
Other background	0	0.0	6	0.4	–	
Relationship status					Overall *p* value = 0.275	
Married	2	1.0	18	1.3	Ref	
Separated	27	13.0	138	9.8	2.13 (0.44 to 10.30), 0.346	
Closely involved/boyfriend	156	75.0	1066	75.6	1.46 (0.32 to 6.78), 0.627	
Just friends	23	11.0	188	13.3	1.20 (0.24 to 5.87), 0.823	
Live with father of baby						
No	127	68.3	985	76.1	Ref	
Yes	59	31.7	309	23.8	1.32 (0.94 to 1.88), 0.113	
*Missing*	*22*		*116*			
Family subjective social status	207	5.4 (5.0 to 7.0)	1400	5.6 (5.0 to 7.0)	***0.90 (0.83 to 0.99), 0.022***	1.01 (0.91 to 1.12), 0.879
Personal subjective social status	208	6.7 (5.0 to 8.0)	1403	6.9 (6.0 to 8.0)	***0.92 (0.85 to 1.00), 0.041***	0.96 (0.88 to 1.05), 0.386
NEET^c^:						
No	89	53.9	720	59.3	***Ref***	
Yes	76	46.1	495	40.7	***1.41 (1.00 to 1.98), 0.050***	
*Not applicable*	*43*		*195*			
Receive any benefits						
No	112	53.8	920	65.3	***Ref***	***Ref***
Yes	96	46.2	488	34.7	***1.61 (1.19 to 2.18), 0.002***	***1.60 (1.09 to 2.35), 0.016***
*Missing*	*0*		*2*			
Ever been homeless						
No	141	67.8	1163	82.5	***Ref***	Ref
Yes	67	32.2	247	17.5	***2.08 (1.49 to 2.91), < 0.001***	1.50 (0.99 to 2.28), 0.059
Socio-economic status: Index of Multiple Deprivation Score^d^	207	39.1 (24.5 to 51.6)	1399	39.2 (25.2 to 52.1)	1.01 (1.00 to 1.02), 0.188	
EQ5D-Binary						
Less than perfect health	97	46.6	488	34.7	***Ref***	Ref
Perfect health	111	53.4	919	65.3	***0.70 (0.51 to 0.95), 0.023***	0.94 (0.63 to 1.38), 0.739
*Not answered*	*0*		*3*			
Self-rated health					Overall *p* value = 0.414	
Excellent	28	13.5	235	16.7	Ref	
Good	143	68.8	942	66.8	1.23 (0.79 to 1.91), 0.355	
Fair	32	15.4	220	15.6	1.17 (0.67 to 2.04), 0.573	
Poor	5	2.4	13	0.9	2.61 (0.82 to 8.27), 0.103	
Limiting long-term illness:						
No	160	76.9	1179	83.6	***Ref***	Ref
Yes	48	23.1	231	16.4	***1.38 (0.96 to 2.00), 0.086***	1.17 (0.77 to 1.77), 0.460
Generalized Self-efficacy Scale^e^	204	29.2 (27.0 to 32.0)	1388	30.1 (28.0 to 33.0)	***0.95 (0.92 to 0.98), 0.002***	
Adaptive functioning^f^ Difficulty in at least 1 basic skill						
No	137	65.9	1048	74.5	**ref**	Ref
Yes	71	34.1	359	25.5	***1.70 (1.23 to 2.35), 0.001***	1.36 (0.94 to 1.96), 0.103
*Missing*	*0*		*3*			
Adaptive functioning^f^ Had 3 or less life skills (out of 5)						
No	157	75.8	1021	72.7	Ref	
Yes	50	24.2	384	27.3	0.92 (0.65 to 1.31), 0.648	
*Missing*	1		5			
Adaptive functioning^f^ At least 1 burden						
No	134	65.0	997	71.2	Ref	
Yes	72	35.0	404	28.8	1.25 (0.91 to 1.72), 0.172	
*Missing*	1		9			
Substance abuse^g^	199	1.6 (0.0 to 3.0)	1399	1.3 (0.0 to 2.0)	***1.23 (1.02 to 1.24), 0.014***	*0.93 (0.83 to 1.05), 0.233*
Antisocial behaviour Score	205	3.1 (2.0 to 4.0)	1404	2.3 (1.0 to 4.0)	***1.30 (1.19 to 1.42), < 0.001***	***1.22 (1.09 to 1.36), < 0.001***
Social support	205	81.5 (73.7 to 96.1)	1398	85.4 (77.6 to 98.7)	***0.99 (0.98 to 0.996), 0.003***	*1.00 (0.99 to 1.01), 0.969*
Relationship quality	171	26.9 (24.0 to 31.0)	1106	28.2 (26.0 to 32.0)	***0.94 (0.91 to 0.97), < 0.001***	*Not included*^*h*^
Family resources	203	12.4 (9.5 to 15.0)	1348	13.5 (11.0 to 17.0)	***0.93 (0.90 to 0.97), < 0.001***	*0.96 (0.92 to 1.00), 0.063*
Psychological distress/mental health	207	23.0 (18.0 to 27.0)	1402	21.3 (16.0 to 26.0)	***1.03 (1.01 to 1.06), 0.003***	*1.01 (0.98 to 1.04), 0.585*
Arm						
Control	100	48.1	710	50.4	Ref	
Intervention	108	51.9	700	49.6	1.10 (0.81 to 1.48), 0.545	

^a^OR predicts a Social AE and adjusted for site; ^b^Adjusted for site and all other candidate predictors in model; ^c^Definition of Not in Education, Employment, or Training (NEET): not in education employment or training status (applicable only to those whose academic age is > 16 years at baseline interview); ^d^Higher IMD score indicated more deprivation; ^e^Higher score indicates higher level of self-efficacy; ^f^Higher score indicates better management of day-to-day lives and routines (for each of the three sub-scales); ^g^CRAFFT screening test for substance related risks and problems in adolescents; ^h^ due to question only applicable to those in a relationship

Odds ratios (ORs) bolded and in italics indicate variable significant at 10% at univariable level and remained so at multivariable level

**Table 6:** Baseline characteristics of participants subsequently with and without at least 1 Medical adverse event (AE) or serious adverse event (SAE)

	At least 1 Medical SAE/AE *N* = 532		Participants without a Medical SAE/AE *N* = 1086		Unadjusted OR^a^ (95% CI), *p* value	Adjusted OR^b^ (95% CI), *p* value
Baseline characteristic	*n*	Median (25th to 75th centile) or %	*n*	Median (25th to 75th centile) or %		
Maternal age (years)	532	17.9 (16.9 to 18.9)	1086	17.8 (16.9 to 18.8)	1.05 (0.97 to 1.15), 0.226	
Ethnicity					*Overall p value = 0.240*	
White background	473	88.9	952	87.7	Ref	
Mixed background	34	6.4	55	5.1	1.45 (0.90 to 2.36), 0.130	
Asian background	12	2.3	15	1.4	1.69 (0.68 to 4.22), 0.259	
Black background	11	2.1	60	5.5	0.65 (0.30 to 1.39), 0.265	
Other background	2	0.4	4	0.4	1.59 (0.27 to 9.45), 0.609	
Relationship status					*Overall p value = 0.847*	
Married	8	1.5	12	1.1	Ref	
Separated	51	9.6	114	10.5	0.73 (0.26 to 2.06), 0.557	
Closely involved/boyfriend	407	76.5	815	75.0	0.67 (0.32 to 1.80), 0.426	
Just friends	66	12.4	145	13.4	0.67 (0.24 to 1.88), 0.448	
Live with father of baby						
No	349	71.8	763	76.8	Ref	
Yes	137	28.2	231	23.2	1.16 (0.88 to 1.52), 0.285	
*Missing*	46		92			
Family subjective social status	527	5.7 (5.0 to 7.0)	1080	5.7 (5.0 to 7.0)	1.00 (0.93 to 1.07), 0.916	
Personal subjective social status	529	6.8 (6.0 to 8.0)	1082	6.9 (6.0 to 8.0)	0.98 (0.92 to 1.04), 0.439	
NEET^c^						
No	279	61.0	530	57.4	ref	
Yes	178	39.0	393	42.6	0.98 (0.76 to 1.26), 0.869	
Not applicable	75		163			
Receive any welfare benefits						
No	346	65.0	686	63.3	Ref	
Yes	186	35.0	398	36.7	1.02 (0.81 to 1.30), 0.848	
*Missing*	0		2			
Ever been homeless						
No	419	78.8	885	81.5	Ref	
Yes	113	21.2	201	18.5	1.24 (0.93 to 1.64), 0.146	
Socio-economic status: Index of Multiple Deprivation Score^d^	528	39.1 (25.9 to 51.3)	1078	39.2 (24.8 to 52.6)	**1.01 (0.99 to 1.01), 0.078**	1.01 (0.998 to 1.01), 0.106
EQ5D-Binary						
Less than perfect health	220	41.4	365	33.7	***Ref***	Ref
Perfect health	312	58.6	718	66.3	***0.73 (0.58 to 0.93), 0.012***	0.98 (0.74 to 1.29), 0.881
Not answered	0		3			
Self-rated health					*Overall p-value = 0.141*	
Excellent	86	6.2	177	16.3	Ref	
Good	345	64.8	740	68.1	1.00 (0.73 to 1.36), 0.985	
Fair	92	17.3	160	14.7	1.20 (0.81 to 1.79), 0.359	
Poor	9	1.7	9	0.8	2.84 (1.02 to 7.95), 0.046	
Limiting long-term illness:						
No	416	78.2	923	85.0	***Ref***	***ref***
Yes	116	21.8	163	15.0	***1.53 (1.14 to 2.06), 0.005***	***1.37 (1.01 to 1.88), 0.046***
Generalized Self-efficacy Scale^e^	526	30.0 (27.0 to 33.0)	1066	30.0 (28.0 to 33.0)	1.00 (0.97 to 1.02), 0.869	
Adaptive functioning^f^ Difficulty in at least 1 basic skill						
No	372	70.3	813	74.9	***ref***	Ref
Yes	157	29.7	273	25.1	***1.38 (1.07 to 1.78), 0.013***	1.18 (0.91 to 1.54), 0.219
*Missing*	3		0			
Adaptive functioning^f^ Had 3 or less life skills (out of 5)						
No	387	72.9	791	73.2	ref	
Yes	144	27.1	290	26.8	1.11 (0.87 to 1.43), 0.404	
*Missing*	1		5			
Adaptive functioning^f^ At least 1 burden						
No	354	67.0	777	72.0	***Ref***	Ref
Yes	174	33.0	302	28.0	***1.26 (0.98 to 1.61), 0.069***	1.09 (0.84 to 1.41), 0.543
*Missing*	3		7			
Substance abuse^g^	513	1.3 (0.0 to 2.0)	1025	1.4 (0.0 to 2.0)	0.98 (0.91 to 1.06), 0.612	
Antisocial behaviour Score	528	2.4 (1.0 to 4.0)	1081	2.4 (1.0 to 4.0)	***1.06 (0.99 to 1.13), 0.089***	1.01 (0.95 to1.09), 0.689
Social support	526	85.1 (77.6 to 98.7)	1077	84.8 (77.6 to 98.7)	1.00 (0.99 to 1.01), 0.784	
Relationship quality	428	28.4 (26.0 to 32.0)	849	28.8 (25.0 to 31.0)	1.00 (0.98 to 1.03), 0.825	
Family resources	510	13.3 (11.0 to 16.0)	1041	13.4 (11.0 to 17.0)	0.98 (0.95 to 1.01), 0.145	
Psychological distress/mental health	529	22.4 (17.0 to 27.0)	1080	21.1 (16.0 to 25.0)	***1.04 (1.02 to 1.06), < 0.001***	***1.03 (1.01 to 1.05), 0.004***
Arm						
Control	243	45.7	567	52.2	***Ref***	***Ref***
Intervention	289	54.3	519	47.8	***1.32 (1.05 to 1.65), 0.016***	***1.34 (1.07 to 1.70), 0.012***

^a^OR predicts a Social AE and adjusted for site; ^b^Adjusted for site and all other candidate predictors in model; ^c^Definition of NEET: Not in Education Employment or Training Status (applicable only to those whose academic age is > 16 years at baseline interview); ^d^Higher IMD: Index of Multiple Deprivation score indicated more deprivation; ^e^Higher score indicates higher level of self-efficacy; ^f^Higher score indicates better management of day-to-day lives and routines (for each of the three sub-scales); ^g^CRAFFT screening test for substance-related risks and problems in adolescents;

Odds ratios (ORs) bolded and in italics indicate variable significant at 10% at univariable level and remained so at multivariable level

Numerous baseline characteristics were identified to be associated with Social AEs including younger mothers, lower family and personal subjective social status, NEET, being in receipt of benefits, homelessness, lower self-efficacy and social support, difficulty in at least one basic skill, lower quality of life, having a limiting long-term illness, more likely to have substance abuse, antisocial behaviour, lower relationship quality and family resources and worse psychological distress (Table 5). No multi-collinearity was found between any of the candidate predictors in the multivariable model (VIF = 1.26). Three predictors were found to be independently associated based on a significance level of 0.05 with Social AEs after adjusting for all other candidate predictors. Participants with at least one Social AE were *more* likely to be younger at recruitment (odds ratio (OR) = 0.78 (CI = 0.67 to 0.90), *p* = 0.001), to receive welfare benefits (OR = 1.60 (CI = 1.09 to 2.35), *p* = 0.016), and have a higher score on a measure for antisocial behaviour (OR = 1.22 (CI = 1.09 to 1.36), *p* < 0.001) (Table 5).

For Medical S/AEs, fewer predictors were apparent at univariable level including a higher deprivation score, less than perfect health, a limiting long-term illness, difficulty in at least one basic skill and having at least one adaptive functioning burden, antisocial behaviour, more psychological distress and randomised to receive FNP (Table 6). Again no collinearity was found between any of the candidate predictors in the multivariable model (VIF = 1.09). Three predictors of Medical S/AEs remained based on a significance level of 0.05 after adjusting for all other candidate predictors in the model (Table 6).

Participants with at least one Medical S/AE were *more* likely to have a limiting long-term illness (OR = 1.37 (CI = 1.01 to 1.88), *p* = 0.046), were more likely to score higher on a measure of psychological distress/mental health (OR = 1.03 (CI = 1.01 to 1.05), *p* = 0.004), and were more likely to be in the intervention arm of the trial (OR = 1.34 (CI = 1.07 to 1.70), *p* = 0.012).

Missing data was limited as baseline trial data was well completed (apart from two variables; NEET and relationship quality) and these were omitted from the multivariable analyses.

## Discussion

Most AEs reported to the Building Blocks trial were classified as being Medical SAEs or AEs of a physical nature. However, our finding that over 19% of events were Social AEs supports the idea that the GCP definition of AEs and SAEs cannot capture all events related to well-being and social circumstances that might be important for a public health or social care trial.

**Fig. 2:**
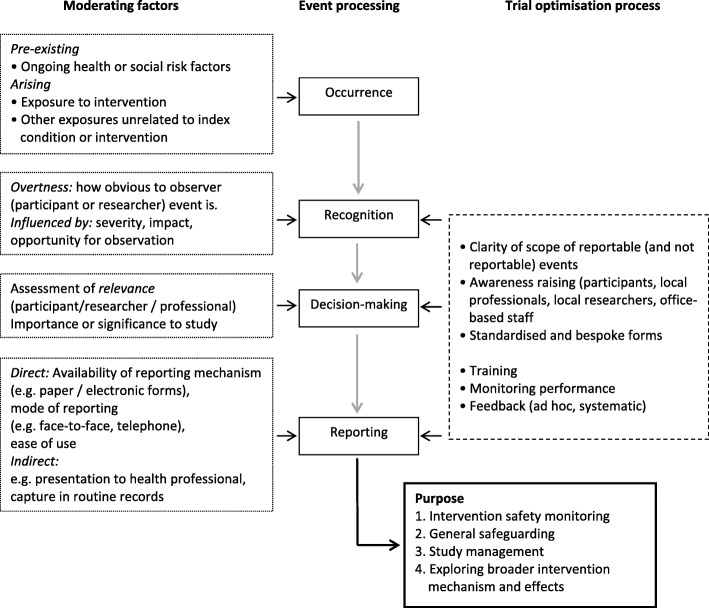
A proposed model of adverse event presentation and reporting

Reporting of AEs in trials requires a number of inter-related processes to occur (Fig. 2). First, there has to be a reportable event; therefore, an ‘event’ needs to be defined. Pre-existing factors related to the individual may affect this; for example, ongoing or intermittent ill-health which may or may not be related to the individual’s trial eligibility. Factors arising during the course of the trial will also affect this, perhaps most notably, but not solely, exposure to the intervention. Second, events need to be recognised as reportable, either by the individual participant or by a relevant professional. The pivotal factors at this stage are how observable the event is, and its severity. Third, a decision needs to be made to formally report. This may involve decision-making by the participant as well as a professional and key to this will be an assessment of relevance (i.e. is the event of sufficient importance?). This is of course a judgement that can be dependent on many factors; e.g. value placed on the particular event, and is it within the scope of interest of the trial? Most of this is pre-defined. Finally, a mechanism needs to facilitate capture of the event. As we have seen in our trial, mechanisms for capture include direct reporting (e.g. to field or office-based researchers using standardised forms), identification through review of routine records, or identification via screening questions.

How well a trial system can capture with precision all relevant events will depend on adequate progression through each of the stages described above. Clinical trials of investigational medicinal products, which may be most concerned with reporting serious Medical AEs, may fare better in adequately progressing through these required processes than trials of complex interventions where unexpected and undesirable impacts may be less tangible and arise within a broader social context. Defining undesirable impacts may be more complex in public health or social care trials, and the use of Public and Patient Involvement to assist with definitions may be particularly useful in some cases.

We hypothesised that lower baseline psycho-social status or poorer health status may increase the likelihood of both Medical and Social AEs. This relates to the first step of our model (i.e. pre-existing factors). Participants with existing conditions are more likely to continue or repeat experience of that condition. We also hypothesised that poorer psycho-social status would be a better predictor of Social AEs rather than of Medical AEs or SAEs, and this hypothesis was also supported. Our third hypothesis was that participants with at least one reported AE or SAE, regardless of whether Medical or Social event, are more likely to be in the intervention arm. This relates to the Recognition and Decision-making steps in our model. Women in receipt of intervention were regularly in contact with a health professional who in turn also promoted her access to supportive services. The personal relationship between a participant and her Family Nurse would have meant an increased number of opportunities for observing events, and would also have increased the likelihood of the women disclosing a concern, which they may not have otherwise presented to another health professional or researcher. We found trial arm to be a predictor of Medical S/AE, but not of Social AEs, thus providing partial support for our hypothesis. It is possible that expected social concerns may have simply been addressed within the routine remit of the Family Nurse’s work, rather than being documented or reportable as a trial AE. Our final hypothesis was that site-level differences would affect reporting of both Medical S/AEs and Social AEs. While we have been unable to fully explore this facet of the process in our analysis, factors that may vary by site cumulatively impact upon successive stages of event processing and are discussed more fully below. These factors could include local capacity, experience of field researchers and adequacy of training cascaded to local professional and research staff. Table 7 summarises our hypotheses in relation to our results.

**Table 7:** Results in relation to hypotheses

Hypotheses	Results
Hypothesis 1: poorer psycho-social status and health at baseline will be associated with higher reported rates of both Medical and Social AEs	Main predictors of Social AEs: younger age at recruitment, more likely to receive benefits, having a higher antisocial behaviour score. Main predictors of Medical S/AEs: ill health at baseline (limiting long-term illness and poorer mental health)
Hypothesis 2: poorer psycho-social status at baseline will more likely be associated with Social rather than Medical AEs	Hypothesis supported. Main predictors of Social AEs (related to poorer psycho-social status): younger age at recruitment, more likely to receive benefits. No baseline indicators of poorer psycho-social status remained in the model for the Medical S/AEs
Hypothesis 3: AEs reports will be more likely for those in the trial intervention arm	Hypothesis partially supported. Allocation to the intervention arm of the trial was associated with increased likelihood of Medical S/AE but not with Social AE reporting
Hypothesis 4: rate of AE reporting will vary by trial site	Partially supported although we have been unable to fully explore this

*AE* adverse event, *SAE* serious adverse event

How trial teams can optimise the capture of AEs is represented to the right of our model (Fig. 2). These include well-established practices such as having a clearly defined set of criteria for reportable events, awareness-raising amongst key stakeholders and provision of accessible reporting forms. While for clinical trials of medical interventions, the scope of reportable events is well established, this will need to be expanded for trials of complex public health and social care interventions. Adherence to these processes will need to be supported through training, performance monitoring and feedback mechanisms which could involve one-to-one review of reported events and/or systematic assessment of sets of reported events. These combined processes are most likely to impact upon the Recognition, Decision-making and Reporting stages of the model.

Taking our trial as an example, process optimisation would involve training field and office-based researchers to ensure that AEs were *collected* in a standardised manner. It is important to collect AE data in a standardised manner to enable researchers to pool evidence from large trials [3], and standardisation also allows researchers to compare efficacy outcomes with AEs reported. There were some variations reported in the ways that AEs were collected in the Building Blocks trial, and this may have had a bearing on the proportion of AEs collected from each site. While advice was provided at the outset about what was reportable as an AE (i.e. a clear definition of what an event is), we revised this advice based on early experiences in the trial. Researchers were responsible for asking local health professional teams; for example, Family Nurses, to alert them to any AEs concerning Building Blocks participants. Stickers were also placed inside participant hospital notes alerting hospital staff to contact the researcher with details of any AEs. Having accessible reporting forms and other guidance defining what is reportable is key. Even though field and office-based researchers were trained in the collection of AEs, verbal reports alluded to some variation in the way AEs that were collected in practice. Some researchers reviewed hospital notes for AEs when collecting data for the birth data collection phase of the trial. While this was valuable for identifying some otherwise unreported events, clearer direction at the outset to target this activity would have reduced some apparent unhelpful variation by site. Scoring positively for items indicating serious abuse on a domestic abuse scale also triggered the completion of an AE form, and formally triangulating between data sources to identify AEs where possible might be another way to improve the collection of AEs. It should also be noted that some events have a subjective element; for example, events related to mental health are probably more subjective than those relating to physical health, and the recognition of an event may be affected by the subjectivity of that event. Other researchers have written about the importance of systematic collection of events in medical trials to produce reliable data [14] and to prevent biased reporting [15]. The subjectivity of medical events may be a reason for the slightly lower agreement during classification when compared to physical events which include more objectively observable physical descriptions symptoms/signs/diagnoses, and for AEs (rather than SAEs) the rôle of subjective decision-making may be greater as the apparent importance is less severe. In this study, although reporting systems for SAEs were systematic (i.e. using a common reporting form) and reporting came via multiple routes, there was not a wholly systematic process for their identification. Ensuring that data was collected in a more systematic way could have been done in a number of ways in the current trial. For example, we could have asked researchers to *all* either periodically search notes for AEs (this was done by a proportion of researchers) or to do this at the end of the trial. While domestic violence was systematically screened for at the end of the study period and where applicable reported as an SAE, other items specifically designed to collect AE data could have been included in the various data collection stages. Tools such as MedDRA have been used for safety monitoring in drug trials; something similar could be used, with supplementary items designed to capture social events. These amendments, however, would have increased costs and participant burden and doing so would have to be balanced against the risk of missing such harms.

Improvements could also have been made in the training given to field and office-based researchers to ensure that AE forms were *completed* in a standardised manner. The quality of an individual case safety report is dependent on the accuracy and completeness of the information gleaned about the case [16], and the same can be said in the case of AE reporting in the Building Blocks trial. The need for training on the completion of a form should be balanced with ensuring that the forms are self-explanatory as many health professionals completing the forms will be doing so without receiving any formal training. For example, as well as field and office-based researchers, other health professionals and even participants may provide information on AEs in the trial. Guidance on determining expectedness of events was provided during the training; however, some events reported as being ‘unexpected’ were subsequently reclassified due to the context of the Building Blocks trial.

Horigian et al. [17] listed five principles for defining AEs in behavioural research and our own study can be viewed in light of these. Firstly, that they should be grounded in previous research, and secondly, queries on AEs should include domains plausibly affected by the interventions being tested. The current study also defined AEs in light of research, but were more open in what were accepted as AE reports. This may have caused some problems with too much interpretation by Research Nurses and too much variation in reporting by site; this issue was responded to with more training. Perhaps a framework of possible AEs should be put in place a priori which then allows for unanticipated AEs to be observed and reported. Compared to some psycho-therapeutic settings, home-visiting is a more complex intervention, may impact on a broader range of outcomes and not solely for the participant (for example, there could be an impact on a partner, parent, etc.). Even though a logic model and previous literature can inform in advance what AEs may be likely, some flexibility within an overarching framework is helpful. Thirdly, monitoring should attempt to assess relatedness between interventions and AEs, we agree with this but it should be kept in mind that relatedness is perhaps even harder to establish when an intervention is delivered over such a long period of time (2.5 years) and where the intervention (in this case FNP) is also seeking to engage the client with a range of other services, social and family support, this simply adds to the complexity of causation. We agree with both the fourth principle that systematic monitoring is essential for identifying unexpected events, and the fifth, that effective monitoring is a shared responsibility. In summary, the current piece of work provides support for Horigian’s model in a different setting (community-based public heath within families of young children). As they comment on the need for the utility of the principles on other settings in their paper, we provide some evidence of that generalisability. A robust theory to identify broad AE domains, in addition to more specific AEs, is essential to capture unexpected AEs, and that training is even more essential to ensure that. Our study provides an example of where we aimed to capture AEs, specifically Medical or Social, although the approach of Horigian et al (2010) would probably actually address both. The approach to monitoring AEs in social and public health is still limited and variable; our study perhaps identifies the need to better train staff to monitor this in more complex intervention settings rather than with clinical patients.

### Strengths and limitations

We have developed a simple classification scheme for monitoring reports of AEs, which explicitly accommodates social as well as medical events. This has been developed over the course of an ongoing trial and, therefore, benefits from review and assessment of actual reports rather than hypothetical examples. Constructing the classification has benefited from the input of the trial team tasked with AE monitoring (including clinical input) which has also been involved in training research staff in collating reports in the field. The experience of discussing the purpose and practice of AE monitoring with this specific trial population has helped to clarify the purpose and scope of event monitoring. While the classification reflects a particular public health intervention and trial population, it nevertheless provides an example of how the existing GCP standard approach to reporting Medical AEs can be expanded to reflect the needs of a specific trial. Finally, while our classification distinguished reliably between Medical and Social AEs, a small number of ‘Other events’ were categorised as neither and excluded from further analysis. It is possible that further details of the reported event or further review of the report received would have resulted in reclassification as either a Medical or a Social event. However, it is probable that other circumstances for trial participants would still be of some logistical or clinical value and, therefore, important to monitor.

The presented analysis benefited from a large sample which was well characterised at baseline, and dependent outcomes produced following a reliable coding process. Our examination of predictors was limited by the large number of levels for the ‘principal site’ variable. Therefore, we are unable to conclude whether apparent variation in reporting by sites could have been due to differences in trial participants between sites, or due to site-level factors such as the local researcher. Given the large variation in event reporting rates between sites with similarly sized participant samples it seems likely that non-participant-related factors are likely to be influencing reporting rates. This is important as it would represent unhelpful noise in an attempt by investigators to accurately monitor safety and well-being for trial participants.

## Conclusions

Active systematic safety monitoring in public health and social care trials which additionally focus on Social AEs is rarely reported. In public health and social care trials, it is likely that there will be adverse experiences that are not medical but may reflect social circumstances. A system of safety monitoring should be considered which would include both Medical and Social AEs. We recognise that this may result in a valid decision not to actively monitor AEs based, for example, on likely frequency and severity. Collecting social events needs to be tailored to the circumstances of the trial and to reflect how the information is likely to be used. This could include assessing any unexpected adverse consequences of the intervention, more general safeguarding of participant well-being during a trial, identifying matters that need to be considered in running the trial (e.g. to avoid contacting participants in distress) and also exploring more broadly the mechanism and broader impacts of an intervention (Fig. 2). How information about AEs will be used should be clearly stated by researchers and guide decision-making about how best to resource and support high-quality data capture.

## Data Availability

The datasets used and/or analysed during the current study are available from the corresponding author on reasonable request but which would require additional processing to ensure confidentiality.
